# Mixing of propagules from discrete sources at long distance: comparing a dispersal tail to an exponential

**DOI:** 10.1186/1472-6785-6-3

**Published:** 2006-02-20

**Authors:** Etienne K Klein, Claire Lavigne, Pierre-Henri Gouyon

**Affiliations:** 1 Unité de Recherches Forestières Méditerranéennes – Unité de Biométrie, Institut National de la Recherche Agronomique, Domaine St-Paul, Site Agroparc, 84914 Avignon cedex 9, France; 2 Unité Plantes et Systèmes de Culture Horticoles, Institut National de la Recherche Agronomique, Domaine St-Paul, Site Agroparc, 84914 Avignon cedex 9, France; 3 Muséum National d'Histoire Naturelle, Département de Systématique & Évolution, Botanique, 12 rue Buffon 75005 Paris CP 39, France

## Abstract

**Background:**

Rare long distance dispersal events impact the demography and the genetic structure of populations. When dispersal is modelled via a dispersal kernel, one possible characterisation of long-distance dispersal is given by the shape of the tail of the kernel, i.e. its type of decay. This characteristic is known to directly act on the speed and pattern of colonization, and on the spatial structure of genetic diversity during colonization. In particular, colonization waves behave differently depending on whether the kernel decreases faster or slower than an exponential (i.e. is thin-tailed vs. fat-tailed). To interpret and extend published results on the impact of long-distance dispersal on the genetic structure of populations, we examine a classification of dispersal kernels based on the shape of their tails and formally demonstrate qualitative differences among them that can influence the predicted diversity of a propagule pool sampled far from two distinct sources.

**Results:**

We show that a fat-tailed kernel leads asymptotically to a diverse propagule pool containing a balanced mixing of the propagules from the two sources, whereas a thin-tailed kernel results in all propagules originating from the closest source. We further show that these results hold for biologically relevant distances under certain circumstances, and in particular if the number of propagules is large enough, as would be the case for pollen or seeds.

**Conclusion:**

To understand the impact of long-distance dispersal on the structure and dynamics of a metapopulation, it might be less important to precisely estimate an average dispersal distance than to determine if the tail of the dispersal kernel is fatter or thinner than that of an exponential function. Depending solely on this characteristic, a metapopulation will behave similarly to an island model with a diverse immigrant pool or to a stepping-stone model with migrants from closest populations. Our results further help to understand why thin-tailed dispersal kernels lead to a colonization wave of constant speed, whereas fat-tailed dispersal kernels lead to a wave of increasing speed. Our results also suggest that the diversity of the pollen cloud of a mother plant should increase with increasing isolation for fat-tailed kernels, whereas it should decrease for thin-tailed kernels.

## Background

In plant species, the patterns of gene flow by way of seed and pollen dispersal determines the demographic behaviour of populations, the spatial distribution of neutral and selected genetic diversities, and their evolution. Long-distance dispersal (LDD) is an important characteristic of dispersal events affecting population ecology, species distribution, evolution, and conservation [1-4]. It can be quantified by the proportion of the dispersers present farther than a specified threshold or by a distance beyond which only a small fraction of dispersers are found, e.g. 1% [4]. An alternative formulation of LDD concerns the shape of the tail of the dispersal kernel. This characteristic is known to play a major role in the speed of colonization [5-7], spatial pattern during colonization [8] and genetic structure after spatial expansion [9]. Because of its major role, much effort is put in characterizing this shape of the dispersal tail, both through a better understanding of dispersal mechanisms (mechanistic models, see e.g. [3]) and through better empirical descriptions of observed patterns (empirical models, see e.g. [10,11]). Dispersal kernels are themselves subject to evolution and LDD should generally be selected for under simple assumptions [12,13].

There exists a large 'set of possibilities' when considering dispersal kernels. When addressing long-distance dispersal, it is common to distinguish leptokurtic (kurtosis of the distribution higher than that of a Gaussian with the same variance) versus platykurtic kernels (e.g. [9]). However, as we will show below, kurtosis is a property of the entire distribution and is an insufficient characterisation of the tail for the purpose of predicting long-distance dispersal. When dealing only with the shape of the tail, a first distinction made is whether the functions are exponentially bounded or not. Functions not exponentially bounded [7] are also named fat-tailed kernels [6]. They can be contrasted with thin-tailed kernels that decrease faster that an exponential function, with an intermediate behaviour for the exponential-like kernels (those that have the same behaviour as the exponential function in ± ∞). Another way of classifying the tails of dispersal kernels is to distinguish whether they are regularly varying or rapidly varying [14]. Roughly speaking (Additional file 1 for a precise definition), power-law decreases, also named algebraic tails, are regularly varying [11] whereas all types of exponential decreases (including exponential-power functions for instance) are rapidly varying.

The way the tail of the dispersal kernel and the number and positions of individuals emitting propagules interact to determine the number of propagules arriving at a long distance is well formalized and documented because it plays a major role in determining the colonization speed (e.g. [5]). The effect of this interaction on the genetic diversity of distant propagule pools and the consequences for the structure of genetic diversity has only been approached through simulations (e.g. [9,15-17]). We investigate here the relative contributions of sources at different distances to the pool of propagules by asking an apparently simple question: at long distance from two isolated propagule sources, does one source dominate in the propagule pool or is there an approximately even mixture of propagules from both sources?

We show that, asymptotically, the answer depends critically on whether the dispersal kernel is fat-tailed or not. Fat-tailed kernels lead to a balanced mixing of the propagules from the two sources, and thus to a diverse propagule pool. Contrarily, thin-tailed kernels result in a propagule pool of low diversity, with nearly all propagules originating from the closest source. We further show that this asymptotic property is valid for biologically relevant distances and numbers of propagules.

## Results and discussion

### Asymptotic results

#### 1-dimension

**Figure 1:**
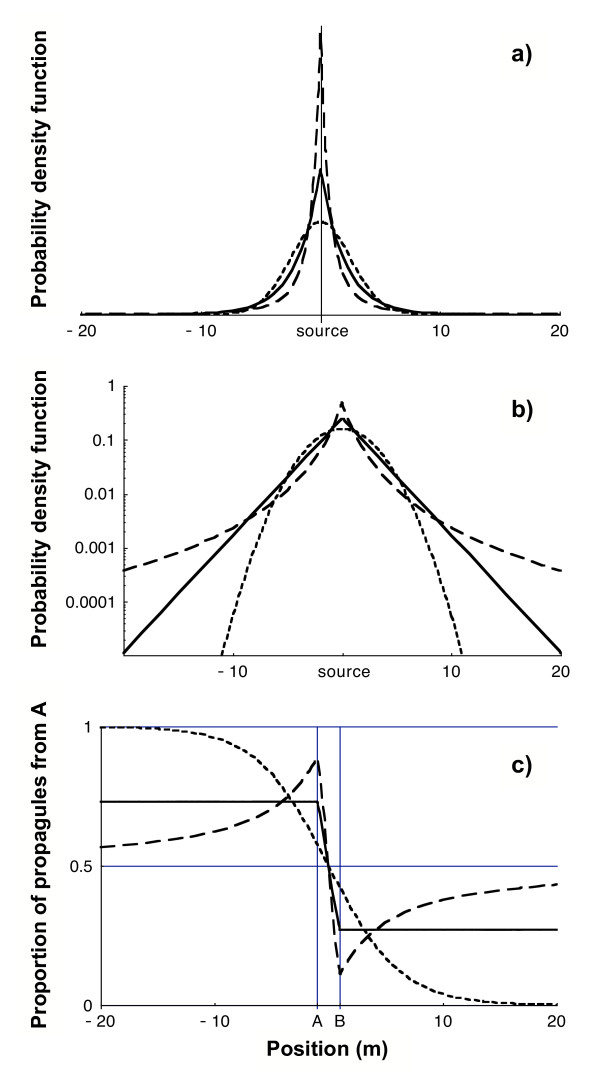
**1-D dispersal kernels and proportion *π*A of propagules from source A **. (*a*) Plot of the three dispersal kernels used (*hatched line*, Gaussian kernel; *solid line*, exponential kernel; *long-hatched line*, power-law kernel with *a *= 3). All three have the same mean distance travelled, equal to 2 m (parameter *α *equals respectively 3.44, 2 and 2). (*b*) Log-plot of the three dispersal kernels (*c*) Proportion of propagules from source A as a function of position *x *between -20 m and 20 m for the three dispersal kernels. The distance between the two sources A and B is 2 m, equal to the mean dispersal distance.

When considering the proportion of propagules originating from each of two sources A and B, in the propagules shadow at point *x*, *π*_A_(*x*) and *π*_B_(*x*), we showed that there is a qualitative difference depending upon the weight of the tail of the dispersal kernel (Methods and Figure 1; In the Additional file 1, we also provide a formal proof general to all families of kernels). Indeed, when going from *x*_*B *_to +∞, the proportion of propagules from A (the farthest source) tends toward:

• 0 for a thin-tailed kernel

• a value *π*_lim _strictly between 0 and 1/2 for an exponential dispersal kernel

• 1/2 for a fat-tailed kernel.

This means that at long distances the propagules from the two sources are well mixed with a ratio approximating 1:1 only if the dispersal kernel is fat-tailed. On the other hand, if the dispersal kernel has a thinner tail than the exponential the fast majority of propagules received originate from the closer source and almost all those originating from the source located farther are absent in the propagule shadow. The exponential kernel thus appears as a critical point where the composition of the propagule shadow changes qualitatively.

**Figure 2:**
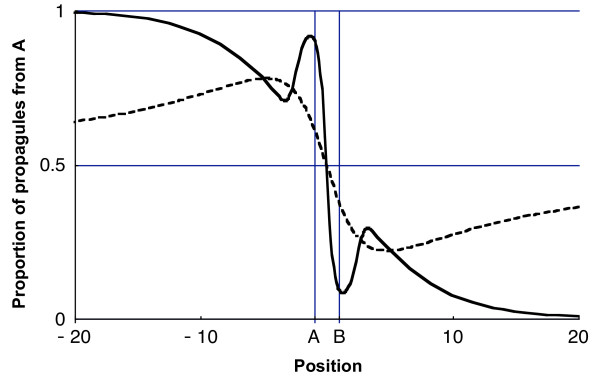
**Proportion *π*A of propagules from source A**. Proportion of propagules from source A as a function of position *x *between -20 m and 20 m for two dispersal kernels:*hatched line*, 1-dimensional 2Dt with *a *= 3 and *α *= 4.7; *solid line*, mixture of two Gaussians with *p *= 2/3, *α*_1 _= 1 and *α*_2 _= 4. The mean distance travelled is the same for both kernels and equals 2 m.

Moreover, for the particular kernels that we studied, we show that the variations in proportions of propagules from A are monotonic between *x*_B _and +∞ with (i) a decrease towards 0 for thin-tailed exponential power kernels, (ii) a constant value for an exponential kernel and (iii) an increase towards 1/2 for fat-tailed exponential power kernels and power-law kernels (Methods and Figure 1). This is unfortunately not the rule for all dispersal kernels suggested. For example, the commonly applied 2Dt model (Table 1 and [18]) or the mixture of two Gaussian (Table 1 and [19]) lead to a function *π*_A_(*x*) tending to the correct asymptotic value (resp. 1/2 and 0) but in non-monotonic ways (Fig. 2). The *π*_A _predicted with the fat-tailed 2Dt model decreases before increasing towards 1/2 and that predicted with the thin-tailed mixture of two Gaussian models increases before decreasing to 0.

#### 2-dimensions

**Figure 3:**
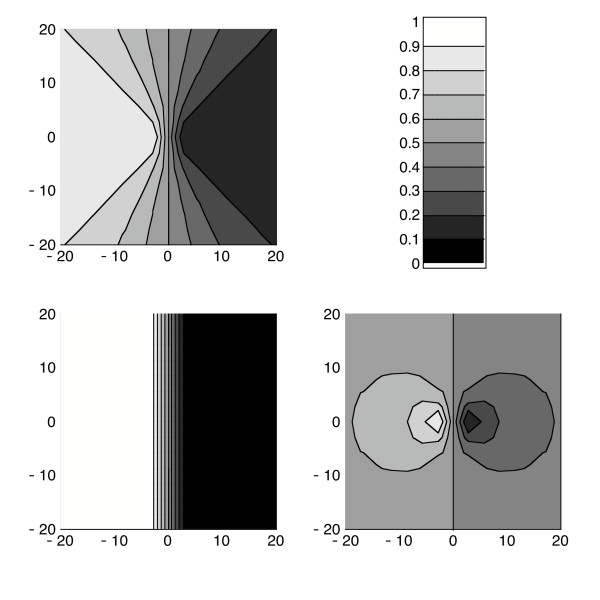
**Proportion *π*A of propagules from source A in a 2-D space**. Proportion of propagules from source A as a function of position (*x, y*) in a 2-dimensional space for three dispersal kernels:*top*, exponential kernel; *bottom left*, Gaussian kernel; *bottom right*, power-law kernel with *a *= 4. The mean distance travelled is the same for the three kernels and equals 2 m (parameter *α *equals respectively 2.26, 1 and 1). The sources A and B are located in (-1,0) and (1,0). The lighter greys stand for larger proportions of propagules from A, such as indicated by the colorbar.

The 1-dimension results are strictly transposable to the 2-dimension problem (Methods), except that the differences in the patterns of mixing are even more striking (Figure 3). When going away from the two sources along a transect, the proportion of propagules from A:

• tends either towards 0 or 1 depending on the direction followed if the kernel is thin-tailed

• tends towards values strictly between 0 and 1, depending on the direction followed, if the kernel is exponential

• tends towards 1/2 independently of the direction followed if the kernel is fat-tailed.

Here again, at long-distances from the two sources, the closest source is the only one that contributes significantly to the propagule shadow for a thin-tailed kernel, whereas both sources evenly contribute for a fat-tailed kernel. The exponential kernel is a critical point between these two behaviours. However, notice that on the line of equidistance between A and B the proportion of propagules from A is logically 1/2, whatever the kernel, and whatever the distance to the sources.

### Finite distances

#### Effect of the range of distances

The asymptotic results presented above are true whatever the distance between the two sources and the mean dispersal distance but only at an infinite distance from the sources. The distances at which this asymptotic behaviour is a good approximation depend on the distance between the sources relative to the mean dispersal distance. The limit value for the exponential kernel, which is between 0 and 1/2, also depends on these two measures.

**Figure 4:**
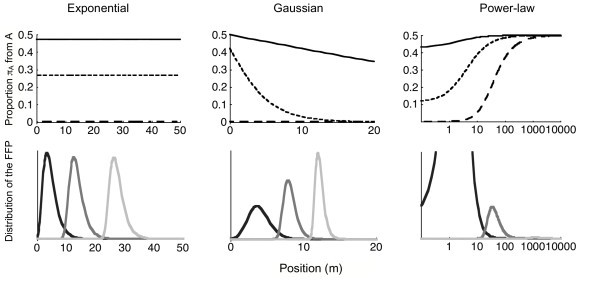
**Proportion *π*A of propagules from source A and position of the FFP from B**. Mixing of propagules as a function of distances to the sources and distances actually travelled by the propagules. For three dispersal kernels (*left*, exponential kernel; *centre*, Gaussian kernel; *right*, power-law kernel with *a *= 3; all having a mean dispersal distance equal to 2 m) we have plotted the proportions of propagules from A as a function of position *x *(*Top*) and the distribution of the further forward propagule from B (*Bottom*). For each kernel, the bottom figure informs on the range of distances where the FFP is expected (for three different amounts of propagules), and thus where the proportion of propagules from A should be read with particular interest on the corresponding top figure. In the top figures, three designs are represented with distance between the sources A and B equal to:*solid line*, 0.2 m; *hatched line*, 2 m; *long- hatched line*, 20 m (the source B is always in 0, and A is located consequently). In the bottom figures, three intensities for the sources A and B have been considered with the amount of propagules emitted *R *equal to: *light gray*, 10^6^; *gray*, 10^3^; *black*, 10. For the different kernels, the *x*-axis scale has been adapted to include the distances actually travelled by propagules, and thus a log-scale has been used for the power-law kernel. When *R *equals 10, 10^3 ^and 10^6 ^the FFP from B is around 5, 15 and 30 m for the exponential, around 4, 8 and 12 m For the Gaussian, and around 1, 30 and 1000 m for the power-law. Thus, for instance, with the power-law kernel the FFP mostly stays within the first 50 m when R = 10 (bottom right, black) where the asymptotic property is only valid for two close sources (top right, solid line). At the opposite, when R = 10^6^, the FFP is likely to travel 1000 m (bottom right, light gray), where the asymptotic is reached whatever the distance between sources (top right, long-hatched line).

When the distance between the two sources is large with regard to the mean distance travelled (that is when (*x*_*B*_*-x*_*A*_)/*δ *is large), the exponential model tends to behave as a thin-tailed kernel (Figure 4, top, hatched lines). Both lead to a negligible proportion of propagules from A in the propagule shadow at the right of source B. For fat-tailed kernels this proportion remains close to 0 in the vicinity of B, but increases as expected to 1/2 farther from B.

When the distance between the two sources is small with regard to the mean distance travelled (that is when (*x*_*B*_*-x*_*A*_)/*δ *is small), the three models tend to behave similarly (Figure 4, top, black lines): the exponential model remains equal to a value close to 1/2, the fat-tailed models increase to 1/2 but starting from a high value at point *x*_*B *_(thus the increase is weak) and the thin-tailed models decrease to 0 very slowly.

#### Range of distances actually travelled in natural conditions

Among a given amount of propagules emitted (*R*), the one that travels the longest distance is known as the furthest forward propagule (FFP). It is of particular importance during a colonisation since its position will define the extent of the population at the next generation. The position of the FFP also indicates the range of distances to consider when evaluating the biological relevance of the asymptotic results given above. As already known [5], the position of this FFP largely depends on the amount of propagules emitted (*R*). This is particularly true for fat-tailed dispersal kernels (Figure 4, bottom).

For the exponential kernel, the proportion of propagules from A in the propagule shadow does not depend on position. Thus, whatever the number of propagules emitted, the level of mixing of propagules only depends on the distances between the sources (relative to the mean distance travelled).

For a thin-tailed kernel the position of the FFP from B shows a weaker dependence to the number of propagules emitted. For the Gaussian we have used the asymptotic behaviour of the propagule shadow (absence of propagules from A) is (i) always reached for far sources, (ii) only reached for large number of propagules emitted (above 10^3^) if the distance between the sources equals the mean dispersal distance and (iii) never reached for nearby sources (Figure 4).

For the power-law kernel we have used, the position of the FFP from B shows huge variations depending on *R*. The asymptotic behaviour of the propagule shadow (one half of the propagules coming from A) is (i) almost always true for close sources, (ii) reached for large number of propagules emitted (above 10^3^) if the distance between the sources equals the mean dispersal distance, and (iii) still observable for far sources if the number of propagules is very large (10^6^).

#### Between the sources

**Figure 5:**
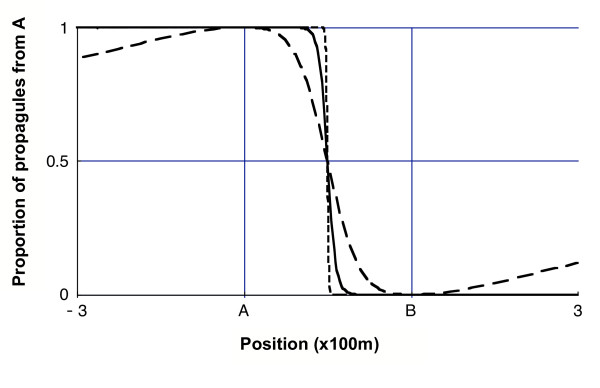
**Proportion of propagules from source A between the sources**. Proportion of propagules from source A as a function of position *x *between -300 m and 300 m for three dispersal kernels:*hatched line*, Gaussian kernel; *solid line*, exponential kernel; *long-hatched line*, power-law kernel with *a *= 3. The mean distance travelled is the same for the three kernels and equals 10 m (parameter *α *equals respectively 17.7, 10 and 10).

The absence of mixing of the propagules from the two sources, observed at long distances for a thin-tailed kernel, can also be seen in the region between the two sources when the distance between the sources is large with regard to the mean distance travelled (high values of the ratio (*x*_*B*_*-x*_*A*_)/*δ*). In particular, the Gaussian model rapidly leads to *π*_*A *_being a binary function equal to 1 if the closer source is A and 0 elsewhere (Figure 5). This means that a point *x *receives almost only propagules from the closer source. Contrarily, fat-tailed kernels lead to functions *π*_*A *_with a smoother decline of *π*_*A *_between *x*_*A *_and *x*_*B*_, thus providing a range of positions (around the origin in Figure 5) where the propagule shadow is diverse.

## Conclusion

Our results contradict a very intuitive idea. One would expect that the propagule shadow received at long distance from two close and distinct sources should be similar to that received from one source emitting half of each propagule type. However, we show that this is only the case if propagules are dispersed following a fat-tailed dispersal kernel: at long distances, a mixing of the propagules from the two sources happens with a ratio 1:1 of propagules from each source. On the other hand, in species with thin-tailed dispersal kernels all the propagules collected far from the sources originate from the closest source, independently of the distance between the sources. Propagules from the farther source are absent in the propagule shadow.

This property has several implications in terms of the dynamics of the genetic diversity over a landscape with several distinct propagule sources. The genetic composition of the propagule pool will qualitatively depend on the type of dispersal kernel. In general, pools are expected to be diverse and little differentiated with fat-tailed dispersal kernels, where all the sources contribute significantly to the pool. The opposite is expected with thin-tailed kernels, where only the closest source has a significant contribution to each pool. In a metapopulation context, our result suggests that a metapopulation will follow an island model or gene pool model [20,21] with fat-tailed kernels, whereas thin-tailed dispersal kernels lead to stepping-stone or one-donor models [20].

Our results further help us to understand why thin-tailed dispersal kernels lead to a colonization wave of constant speed, whereas fat-tailed dispersal kernels lead to a wave of increasing speed [5,22,23]. With thin-tailed dispersal kernels the only individuals/sources that contribute to the advance of the front of the wave are those already located on the front; and the number of these individuals/sources remains constant. On the contrary, with fat-tailed dispersal kernels, all the individuals/sources in the population contribute to the colonization events. Thus, as the population grows so does the number of long distance dispersal events as well as the longest distance travelled. The speed of advance of the wave thus increases. Investigating the position of the parent of the furthest forward propagule could help to confirm this idea. The consequences of our analysis are less clear concerning the genetic structure during colonization. Fat-tailed kernels lead to long-distance dispersers founding very isolated satellite populations, with large founder effects (e.g. [24]). Our results indicate that all the individuals of the population are putative parents of these long-distance dispersers. Thus concerning the global genetic structure we expect a high spatial differentiation, but with no general isolation by distance pattern, a same genotype being present in very distant positions. For thin-tailed kernels, no long-distance founding events are expected, and only the few individuals in the front of the colonization contribute to the next settlers (which is a typical property of the diffusion models, obtained from Gaussian kernels [8]). This could imply a progressive loss of diversity during the advance of the front, leading to weaker differentiation [15] and an isolation by distance pattern. This thin-tailed scenario is particularly well illustrated by [25] who show that a new variant appearing on the front of a colonization either stays where it appeared or moves with the colonization front but in both cases variant individuals remain clustered. Remark finally that the significance of the foundation events in reducing diversity, particularly for fat-tailed kernels, could be decreased if the further propagules are more clumped than modelled usually. This is indeed a pattern observed in some experiments (e.g. [26]). It though depends on whether the clumped propagules originate from the same source or not.

Obviously, no dispersal kernel will extend forever *stricto sensu *but this does not discredit asymptotic results, as illustrated in many scientific domains. Yet, the validity of the asymptotic results should be checked for ecologically relevant dispersal distances, which we investigated here by considering the position of the further forward propagule. We show here that, in practice, the difference between thin and fat-tailed kernels depends on the interactions among (i) the distance between sources, (ii) the mean dispersal distance or any scale parameter of the dispersal kernel (actually, the ratio of distance between sources to the mean dispersal distance gathers i and ii) and (iii) the number of propagules emitted by the sources. For instance, when sources are very close, the absence of mixture for thin-tailed kernels is only effective at very long distances, whereas it is effective at realistic distances when sources are distant enough. Since the position of the furthest forward propagule increases with the number of propagules dispersed, these realistic distances are more likely to be reached for larger numbers of propagules.

Pollen dispersal is a typical case where large numbers of propagules are dispersed. Our results show that the outcrossing part of the pollen pool of isolated plants will be diverse only if the dispersal kernel is fat-tailed. At the opposite, the contribution of the closest pollen donors will be largely dominant if the kernel is thin-tailed. It thus seems that a correct estimation of the shape of dispersal kernels is even more crucial for pollen than for seed dispersal if we wish to predict the impact of population fragmentation or low density on the maintenance and spatial structure of genetic diversity. Empirical studies that compare thin and fat-tailed kernels estimated with a variety of methods tend to find that pollen dispersal kernels are fat-tailed in tree species [27-29] as well as in grasses and forbs ([30] using paternity analysis; [31] for a review on crops, [32,33] using phenotypic markers). Interestingly, as expected from our results, if pollen dispersal kernels are generally fat-tailed, reasonable low densities or levels of isolation tend to promote diversity of pollen clouds, as estimated by an efficient number of fathers in tree species ([34] for a review) or correlated paternity within sibships [35]. Diverse pollen clouds were observed, for example, over isolated or low density individuals of *Prunus mahaleb *L. [36], *Pinus sylvestris *[35] or *Sorbus torminalis *(Oddou-Muratorio *et al*. submitted). Similar results were obtained on male-sterile plants of oilseed-rape [17]. Among these species, the last three had been shown to disperse pollen following fat-tailed dispersal kernels [17,27,33,37]. Empirical results showing a diversity of pollen pools over reasonably isolated individuals thus tend to indicate that pollen dispersal kernels are generally fat-tailed. A thorough investigation of the impact of the number and spatial arrangement of sources on the results presented in this study would be necessary before drawing definite conclusions.

## Methods

### 1-dimensional asymptotic results

Let us consider two sources of propagules A and B (seeds, pollen or spores), located in a 1-dimensional space at positions *x*_*A *_and *x*_*B*_*>x*_*A*_. Those two sources are assumed to emit the same quantity of propagules and to disperse them around themselves following the same kernel *γ*(*x*). The proportion of propagules from A received at point *x *is independent of the total number of propagules emitted by each source. This can be written

πA(x)=γ(x−xA)γ(x−xA)+γ(x−xB)=11+ρB(x),
 MathType@MTEF@5@5@+=feaafiart1ev1aaatCvAUfKttLearuWrP9MDH5MBPbIqV92AaeXatLxBI9gBaebbnrfifHhDYfgasaacH8akY=wiFfYdH8Gipec8Eeeu0xXdbba9frFj0=OqFfea0dXdd9vqai=hGuQ8kuc9pgc9s8qqaq=dirpe0xb9q8qiLsFr0=vr0=vr0dc8meaabaqaciaacaGaaeqabaqabeGadaaakeaaiiGacqWFapaCdaWgaaWcbaGaemyqaeeabeaakiabcIcaOiabdIha4jabcMcaPiabg2da9maalaaabaGae83SdCMaeiikaGIaemiEaGNaeyOeI0IaemiEaG3aaSbaaSqaaiabdgeabbqabaGccqGGPaqkaeaacqWFZoWzcqGGOaakcqWG4baEcqGHsislcqWG4baEdaWgaaWcbaGaemyqaeeabeaakiabcMcaPiabgUcaRiab=n7aNjabcIcaOiabdIha4jabgkHiTiabdIha4naaBaaaleaacqWGcbGqaeqaaOGaeiykaKcaaiabg2da9maalaaabaGaeGymaedabaGaeGymaeJaey4kaSIae8xWdi3aaSbaaSqaaiabdkeacbqabaGccqGGOaakcqWG4baEcqGGPaqkaaGaeiilaWcaaa@5913@

where ρB(x)=γ(x−xB)γ(x−xA)
 MathType@MTEF@5@5@+=feaafiart1ev1aaatCvAUfKttLearuWrP9MDH5MBPbIqV92AaeXatLxBI9gBaebbnrfifHhDYfgasaacH8akY=wiFfYdH8Gipec8Eeeu0xXdbba9frFj0=OqFfea0dXdd9vqai=hGuQ8kuc9pgc9s8qqaq=dirpe0xb9q8qiLsFr0=vr0=vr0dc8meaabaqaciaacaGaaeqabaqabeGadaaakeaaiiGacqWFbpGCdaWgaaWcbaGaemOqaieabeaakiabcIcaOiabdIha4jabcMcaPiabg2da9maalaaabaGae83SdCMaeiikaGIaemiEaGNaeyOeI0IaemiEaG3aaSbaaSqaaiabdkeacbqabaGccqGGPaqkaeaacqWFZoWzcqGGOaakcqWG4baEcqGHsislcqWG4baEdaWgaaWcbaGaemyqaeeabeaakiabcMcaPaaaaaa@44E1@ is the ratio of the number of propagules from B over the number of propagules from A. If *ρ*_*B *_tends to 0, *π*_*A *_tends to 1, if *ρ*_*B *_tends to 1, *π*_*A *_tends to 1/2 and if *ρ*_*B *_tends to +∞, *π*_*A *_tends to 0. We shall now calculate this ratio for three different types of dispersal kernels (Table 1) chosen because (i) they are the most commonly used in the literature, (ii) they are the simplest parametric families for dispersal kernels and (iii) they can be associated through sums and products to encompass almost all models of dispersal kernels (detailed below).

#### Exponential kernels

For the exponential kernels, the ratio of propagules from source B over propagules from source A has the following form between *x*_*B *_and +∞:

ρB(x)=e(xB−xA)/α>1.
 MathType@MTEF@5@5@+=feaafiart1ev1aaatCvAUfKttLearuWrP9MDH5MBPbIqV92AaeXatLxBI9gBaebbnrfifHhDYfgasaacH8akY=wiFfYdH8Gipec8Eeeu0xXdbba9frFj0=OqFfea0dXdd9vqai=hGuQ8kuc9pgc9s8qqaq=dirpe0xb9q8qiLsFr0=vr0=vr0dc8meaabaqaciaacaGaaeqabaqabeGadaaakeaaiiGacqWFbpGCdaWgaaWcbaGaemOqaieabeaakiabcIcaOiabdIha4jabcMcaPiabg2da9iabdwgaLnaaCaaaleqabaGaeiikaGIaemiEaG3aaSbaaWqaaiabdkeacbqabaWccqGHsislcqWG4baEdaWgaaadbaGaemyqaeeabeaaliabcMcaPiabc+caViab=f7aHbaakiabg6da+iabigdaXiabc6caUaaa@42E6@

This means that *ρ*_*B *_and *π*_*A *_do not depend on *x *for *x*>*x*_*B *_and that *π*_*A *_takes a constant value between 0 and 0.5.

#### Power-law kernels

For power-law kernels, the ratio of propagules from source B over propagules from source A between *x*_*B *_and +∞ equals:

ρB(x)=(1+x−xBα1+x−xAα)−a>1.
 MathType@MTEF@5@5@+=feaafiart1ev1aaatCvAUfKttLearuWrP9MDH5MBPbIqV92AaeXatLxBI9gBaebbnrfifHhDYfgasaacH8akY=wiFfYdH8Gipec8Eeeu0xXdbba9frFj0=OqFfea0dXdd9vqai=hGuQ8kuc9pgc9s8qqaq=dirpe0xb9q8qiLsFr0=vr0=vr0dc8meaabaqaciaacaGaaeqabaqabeGadaaakeaaiiGacqWFbpGCdaWgaaWcbaGaemOqaieabeaakiabcIcaOiabdIha4jabcMcaPiabg2da9maabmaabaWaaSaaaeaacqaIXaqmcqGHRaWkdaWcaaqaaiabdIha4jabgkHiTiabdIha4naaBaaaleaacqWGcbGqaeqaaaGcbaGae8xSdegaaaqaaiabigdaXiabgUcaRmaalaaabaGaemiEaGNaeyOeI0IaemiEaG3aaSbaaSqaaiabdgeabbqabaaakeaacqWFXoqyaaaaaaGaayjkaiaawMcaamaaCaaaleqabaGaeyOeI0IaeeyyaegaaOGaeyOpa4JaeGymaeJaeiOla4caaa@4C03@

When *x *tends to +∞, *ρ*_*B *_tends to 1, and thus *π*_*A *_tends to 1/2. A second property is that *ρ*_*B *_decreases for *x*>*x*_*B *_(this can be checked by writing it in the classical form (x−ξx−ζ)a
 MathType@MTEF@5@5@+=feaafiart1ev1aaatCvAUfKttLearuWrP9MDH5MBPbIqV92AaeXatLxBI9gBaebbnrfifHhDYfgasaacH8akY=wiFfYdH8Gipec8Eeeu0xXdbba9frFj0=OqFfea0dXdd9vqai=hGuQ8kuc9pgc9s8qqaq=dirpe0xb9q8qiLsFr0=vr0=vr0dc8meaabaqaciaacaGaaeqabaqabeGadaaakeaadaqadaqaamaalaaabaGaemiEaGNaeyOeI0ccciGae8NVdGhabaGaemiEaGNaeyOeI0Iae8NTdOhaaaGaayjkaiaawMcaamaaCaaaleqabaGaemyyaegaaaaa@380B@ with *ξ*<*ζ*<0), and consequently, *π*_*A *_increases for *x*>*x*_*B*_.

#### Exponential power kernels

In the case of a kernel from the exponential power family, (including the Gaussian kernels), the ratio of propagules from source B over propagules from source A when *x*>*x*_*B *_is given by

ρB(x)=exp⁡((x−xAα)c−(x−xBα)c),
 MathType@MTEF@5@5@+=feaafiart1ev1aaatCvAUfKttLearuWrP9MDH5MBPbIqV92AaeXatLxBI9gBaebbnrfifHhDYfgasaacH8akY=wiFfYdH8Gipec8Eeeu0xXdbba9frFj0=OqFfea0dXdd9vqai=hGuQ8kuc9pgc9s8qqaq=dirpe0xb9q8qiLsFr0=vr0=vr0dc8meaabaqaciaacaGaaeqabaqabeGadaaakeaaiiGacqWFbpGCdaWgaaWcbaGaemOqaieabeaakiabcIcaOiabdIha4jabcMcaPiabg2da9iGbcwgaLjabcIha4jabcchaWnaabmaabaWaaeWaaeaadaWcaaqaaiabdIha4jabgkHiTiabdIha4naaBaaaleaacqWGbbqqaeqaaaGcbaGae8xSdegaaaGaayjkaiaawMcaamaaCaaaleqabaGaem4yamgaaOGaeyOeI0YaaeWaaeaadaWcaaqaaiabdIha4jabgkHiTiabdIha4naaBaaaleaacqWGcbGqaeqaaaGcbaGae8xSdegaaaGaayjkaiaawMcaamaaCaaaleqabaGaem4yamgaaaGccaGLOaGaayzkaaGaeiilaWcaaa@4F25@

and this function can be written as

ρB(x)=exp⁡(xcαc(cxB−xAx+o(x−1))),
 MathType@MTEF@5@5@+=feaafiart1ev1aaatCvAUfKttLearuWrP9MDH5MBPbIqV92AaeXatLxBI9gBaebbnrfifHhDYfgasaacH8akY=wiFfYdH8Gipec8Eeeu0xXdbba9frFj0=OqFfea0dXdd9vqai=hGuQ8kuc9pgc9s8qqaq=dirpe0xb9q8qiLsFr0=vr0=vr0dc8meaabaqaciaacaGaaeqabaqabeGadaaakeaaiiGacqWFbpGCdaWgaaWcbaGaemOqaieabeaakiabcIcaOiabdIha4jabcMcaPiabg2da9iGbcwgaLjabcIha4jabcchaWnaabmaabaWaaSaaaeaacqWG4baEdaahaaWcbeqaaiabdogaJbaaaOqaaiab=f7aHnaaCaaaleqabaGaem4yamgaaaaakmaabmaabaGaem4yam2aaSaaaeaacqWG4baEdaWgaaWcbaGaemOqaieabeaakiabgkHiTiabdIha4naaBaaaleaacqWGbbqqaeqaaaGcbaGaemiEaGhaaiabgUcaRiabb+gaVnaabmaabaGaemiEaG3aaWbaaSqabeaacqGHsislcqaIXaqmaaaakiaawIcacaGLPaaaaiaawIcacaGLPaaaaiaawIcacaGLPaaacqGGSaalaaa@52D4@

where *o*(*x*^-1^) is any function tending to 0 strictly faster than *x*^-1 ^in +∞. So, in +∞,

ρB(x)∼exp⁡((xB−xA)cxc−1αc).
 MathType@MTEF@5@5@+=feaafiart1ev1aaatCvAUfKttLearuWrP9MDH5MBPbIqV92AaeXatLxBI9gBaebbnrfifHhDYfgasaacH8akY=wiFfYdH8Gipec8Eeeu0xXdbba9frFj0=OqFfea0dXdd9vqai=hGuQ8kuc9pgc9s8qqaq=dirpe0xb9q8qiLsFr0=vr0=vr0dc8meaabaqaciaacaGaaeqabaqabeGadaaakeaaiiGacqWFbpGCdaWgaaWcbaGaemOqaieabeaakiabcIcaOiabdIha4jabcMcaPiablYJi6iGbcwgaLjabcIha4jabcchaWnaabmaabaWaaeWaaeaacqWG4baEdaWgaaWcbaGaemOqaieabeaakiabgkHiTiabdIha4naaBaaaleaacqWGbbqqaeqaaaGccaGLOaGaayzkaaGaem4yam2aaSaaaeaacqWG4baEdaahaaWcbeqaaiabdogaJjabgkHiTiabigdaXaaaaOqaaiab=f7aHnaaCaaaleqabaGaem4yamgaaaaaaOGaayjkaiaawMcaaiabc6caUaaa@4BF2@

Thus, *ρ*_*B *_tends to +∞ if *c*>1 and to 1 if *c*<1. Consequently, *π*_*A *_tends to 0 if *c*>1 and tends to 1/2 if *c*<1. The particular case *c *= 1, corresponding to the exponential kernels leads to the same result as above, i.e. a limit strictly between 0 and 1/2.

Moreover, for *x*>*x*_*B*_, the derivative of *ρ*_*B *_is

ρ′B(x)=cα((x−xAα)c−1−(x−xBα)c−1)ρB(x),
 MathType@MTEF@5@5@+=feaafiart1ev1aaatCvAUfKttLearuWrP9MDH5MBPbIqV92AaeXatLxBI9gBaebbnrfifHhDYfgasaacH8akY=wiFfYdH8Gipec8Eeeu0xXdbba9frFj0=OqFfea0dXdd9vqai=hGuQ8kuc9pgc9s8qqaq=dirpe0xb9q8qiLsFr0=vr0=vr0dc8meaabaqaciaacaGaaeqabaqabeGadaaakeaaiiGacuWFbpGCgaqbamaaBaaaleaacqWGcbGqaeqaaOGaeiikaGIaemiEaGNaeiykaKIaeyypa0ZaaSaaaeaacqWGJbWyaeaacqWFXoqyaaWaaeWaaeaadaqadaqaamaalaaabaGaemiEaGNaeyOeI0IaemiEaG3aaSbaaSqaaiabdgeabbqabaaakeaacqWFXoqyaaaacaGLOaGaayzkaaWaaWbaaSqabeaacqWGJbWycqGHsislcqaIXaqmaaGccqGHsisldaqadaqaamaalaaabaGaemiEaGNaeyOeI0IaemiEaG3aaSbaaSqaaiabdkeacbqabaaakeaacqWFXoqyaaaacaGLOaGaayzkaaWaaWbaaSqabeaacqWGJbWycqGHsislcqaIXaqmaaaakiaawIcacaGLPaaacqWFbpGCdaWgaaWcbaGaemOqaieabeaakiabcIcaOiabdIha4jabcMcaPiabcYcaSaaa@57D9@

which is positive when *c*>1 and negative when *c*<1. Consequently, for *c*>1, *ρ*_*B *_increases towards +∞ when *x *increases if *x*>*x*_*B *_and *π*_*A *_thus decreases towards 0. When *c*<1, *ρ*_*B *_decreases towards 1 when *x *increases if *x*>*x*_*B *_and *π*_*A *_thus increases towards 1/2.

#### Rules for compounds of simple functions

If a kernel *γ*(*x*) can be written as the weighted sum of two simpler functions, *γ*(*x*) = *w*_1_*g*_1_(*x*) + *w*_2_*g*_2 _(*x*), where *g*_2 _has a heavier tail than *g*_1 _(i.e. g2(x)g1(x)→∞
 MathType@MTEF@5@5@+=feaafiart1ev1aaatCvAUfKttLearuWrP9MDH5MBPbIqV92AaeXatLxBI9gBaebbnrfifHhDYfgasaacH8akY=wiFfYdH8Gipec8Eeeu0xXdbba9frFj0=OqFfea0dXdd9vqai=hGuQ8kuc9pgc9s8qqaq=dirpe0xb9q8qiLsFr0=vr0=vr0dc8meaabaqaciaacaGaaeqabaqabeGadaaakeaadaWcaaqaaiabdEgaNnaaBaaaleaacqaIYaGmaeqaaOGaeiikaGIaemiEaGNaeiykaKcabaGaem4zaC2aaSbaaSqaaiabigdaXaqabaGccqGGOaakcqWG4baEcqGGPaqkaaGaeyOKH4QaeyOhIukaaa@3B6C@ when *x *tends to ∞) then the ratio of propagules from source B tends to the same value as that obtained for the function *g*_2 _when *x *tends to ∞.

This can be seen from the ratio ρB(x)=γ(x−xB)γ(x−xA)=w1g1(x−xB)+w2g2(x−xB)w1g1(x−xA)+w2g2(x−xA)
 MathType@MTEF@5@5@+=feaafiart1ev1aaatCvAUfKttLearuWrP9MDH5MBPbIqV92AaeXatLxBI9gBaebbnrfifHhDYfgasaacH8akY=wiFfYdH8Gipec8Eeeu0xXdbba9frFj0=OqFfea0dXdd9vqai=hGuQ8kuc9pgc9s8qqaq=dirpe0xb9q8qiLsFr0=vr0=vr0dc8meaabaqaciaacaGaaeqabaqabeGadaaakeaaiiGacqWFbpGCdaWgaaWcbaGaemOqaieabeaakiabcIcaOiabdIha4jabcMcaPiabg2da9maalaaabaGae83SdCMaeiikaGIaemiEaGNaeyOeI0IaemiEaG3aaSbaaSqaaiabdkeacbqabaGccqGGPaqkaeaacqWFZoWzcqGGOaakcqWG4baEcqGHsislcqWG4baEdaWgaaWcbaGaemyqaeeabeaakiabcMcaPaaacqGH9aqpdaWcaaqaaiabdEha3naaBaaaleaacqaIXaqmaeqaaOGaem4zaC2aaSbaaSqaaiabigdaXaqabaGccqGGOaakcqWG4baEcqGHsislcqWG4baEdaWgaaWcbaGaemOqaieabeaakiabcMcaPiabgUcaRiabdEha3naaBaaaleaacqaIYaGmaeqaaOGaem4zaC2aaSbaaSqaaiabikdaYaqabaGccqGGOaakcqWG4baEcqGHsislcqWG4baEdaWgaaWcbaGaemOqaieabeaakiabcMcaPaqaaiabdEha3naaBaaaleaacqaIXaqmaeqaaOGaem4zaC2aaSbaaSqaaiabigdaXaqabaGccqGGOaakcqWG4baEcqGHsislcqWG4baEdaWgaaWcbaGaemyqaeeabeaakiabcMcaPiabgUcaRiabdEha3naaBaaaleaacqaIYaGmaeqaaOGaem4zaC2aaSbaaSqaaiabikdaYaqabaGccqGGOaakcqWG4baEcqGHsislcqWG4baEdaWgaaWcbaGaemyqaeeabeaakiabcMcaPaaaaaa@7777@, in which the first terms of both the numerator and the denominator will be negligible in regards to the second terms when *x *tends to ∞.

If a kernel *γ*(*x*) can be written as the product of two simpler functions, *γ*(*x*) = *g*_1_(*x*) × *g*_2_(*x*), where *g*_2 _has a heavier tail than *g*_1 _(i.e. g2(x)g1(x)→∞
 MathType@MTEF@5@5@+=feaafiart1ev1aaatCvAUfKttLearuWrP9MDH5MBPbIqV92AaeXatLxBI9gBaebbnrfifHhDYfgasaacH8akY=wiFfYdH8Gipec8Eeeu0xXdbba9frFj0=OqFfea0dXdd9vqai=hGuQ8kuc9pgc9s8qqaq=dirpe0xb9q8qiLsFr0=vr0=vr0dc8meaabaqaciaacaGaaeqabaqabeGadaaakeaadaWcaaqaaiabdEgaNnaaBaaaleaacqaIYaGmaeqaaOGaeiikaGIaemiEaGNaeiykaKcabaGaem4zaC2aaSbaaSqaaiabigdaXaqabaGccqGGOaakcqWG4baEcqGGPaqkaaGaeyOKH4QaeyOhIukaaa@3B6C@ when *x *tends to ∞), then the ratio of propagules from source B behaves, when *x *tends to ∞, as that obtained for the function *g*_1_.

Indeed the ratio *ρ*_*B *_can be written as a product of two ratios:

ρB(x)=γ(x−xB)γ(x−xA)=g1(x−xB)g1(x−xA)×g2(x−xB)g2(x−xA)
 MathType@MTEF@5@5@+=feaafiart1ev1aaatCvAUfKttLearuWrP9MDH5MBPbIqV92AaeXatLxBI9gBaebbnrfifHhDYfgasaacH8akY=wiFfYdH8Gipec8Eeeu0xXdbba9frFj0=OqFfea0dXdd9vqai=hGuQ8kuc9pgc9s8qqaq=dirpe0xb9q8qiLsFr0=vr0=vr0dc8meaabaqaciaacaGaaeqabaqabeGadaaakeaaiiGacqWFbpGCdaWgaaWcbaGaemOqaieabeaakiabcIcaOiabdIha4jabcMcaPiabg2da9maalaaabaGae83SdCMaeiikaGIaemiEaGNaeyOeI0IaemiEaG3aaSbaaSqaaiabdkeacbqabaGccqGGPaqkaeaacqWFZoWzcqGGOaakcqWG4baEcqGHsislcqWG4baEdaWgaaWcbaGaemyqaeeabeaakiabcMcaPaaacqGH9aqpdaWcaaqaaiabdEgaNnaaBaaaleaacqaIXaqmaeqaaOGaeiikaGIaemiEaGNaeyOeI0IaemiEaG3aaSbaaSqaaiabdkeacbqabaGccqGGPaqkaeaacqWGNbWzdaWgaaWcbaGaeGymaedabeaakiabcIcaOiabdIha4jabgkHiTiabdIha4naaBaaaleaacqWGbbqqaeqaaOGaeiykaKcaaiabgEna0oaalaaabaGaem4zaC2aaSbaaSqaaiabikdaYaqabaGccqGGOaakcqWG4baEcqGHsislcqWG4baEdaWgaaWcbaGaemOqaieabeaakiabcMcaPaqaaiabdEgaNnaaBaaaleaacqaIYaGmaeqaaOGaeiikaGIaemiEaGNaeyOeI0IaemiEaG3aaSbaaSqaaiabdgeabbqabaGccqGGPaqkaaaaaa@6D62@. If the first ratio tends to ∞, then *ρ*_*B *_tends to +∞, whatever the limit of the second ratio (because this second ratio is larger than 1). If the first ratio tends to 1, so does the second ratio since *g*_2 _has a heavier tail than *g*_1 _and thus *ρ*_*B *_tends to 1. If the first ratio tends to a limit strictly over 1, then the second ratio tends either to 1 or to a limit strictly over 1 and in both cases, *ρ*_*B *_tends to a limit strictly over 1.

### 2-dimensional asymptotic results

The analyses can readily be extended to the two-dimensional case by considering two point sources of propagules A and B, located at positions (*x*_*A*_, 0) and (*x*_*B*_, 0) with *x*_*A *_= -*x*_*B*_. Those two sources are assumed to emit the same number of propagules and to disperse them around themselves following the same 2-dimensional kernel *γ*(*x, y*). We consider only isotropic kernels, that is kernels satisfying γ(x,y)=γr(x2+y2)
 MathType@MTEF@5@5@+=feaafiart1ev1aaatCvAUfKttLearuWrP9MDH5MBPbIqV92AaeXatLxBI9gBaebbnrfifHhDYfgasaacH8akY=wiFfYdH8Gipec8Eeeu0xXdbba9frFj0=OqFfea0dXdd9vqai=hGuQ8kuc9pgc9s8qqaq=dirpe0xb9q8qiLsFr0=vr0=vr0dc8meaabaqaciaacaGaaeqabaqabeGadaaakeaaiiGacqWFZoWzcqGGOaakcqWG4baEcqGGSaalcqWG5bqEcqGGPaqkcqGH9aqpcqWFZoWzdaWgaaWcbaGaemOCaihabeaakmaabmaabaWaaOaaaeaacqWG4baEdaahaaWcbeqaaiabikdaYaaakiabgUcaRiabdMha5naaCaaaleqabaGaeGOmaidaaaqabaaakiaawIcacaGLPaaaaaa@3FEC@. Each point (*x, y*) where we calculate the proportion of propagules coming from source A can be expressed in polar coordinates by a distance *r *and an angle *θ*. Its distance to point B is then given by

r′=r(cos⁡θ−xB)2+(rsin⁡θ)2=r2−2rcos⁡θxB+xB2,
 MathType@MTEF@5@5@+=feaafiart1ev1aaatCvAUfKttLearuWrP9MDH5MBPbIqV92AaeXatLxBI9gBaebbnrfifHhDYfgasaacH8akY=wiFfYdH8Gipec8Eeeu0xXdbba9frFj0=OqFfea0dXdd9vqai=hGuQ8kuc9pgc9s8qqaq=dirpe0xb9q8qiLsFr0=vr0=vr0dc8meaabaqaciaacaGaaeqabaqabeGadaaakeaacuWGYbGCgaqbaiabg2da9maakaaabaGaemOCaiNaeiikaGIagi4yamMaei4Ba8Maei4CamhcciGae8hUdeNaeyOeI0IaemiEaG3aaSbaaSqaaiabdkeacbqabaGccqGGPaqkdaahaaWcbeqaaiabikdaYaaakiabgUcaRiabcIcaOiabdkhaYjGbcohaZjabcMgaPjabc6gaUjab=H7aXjabcMcaPmaaCaaaleqabaGaeGOmaidaaaqabaGccqGH9aqpdaGcaaqaaiabdkhaYnaaCaaaleqabaGaeGOmaidaaOGaeyOeI0IaeGOmaiJaemOCaiNagi4yamMaei4Ba8Maei4CamNae8hUdeNaemiEaG3aaSbaaSqaaiabdkeacbqabaGccqGHRaWkcqWG4baEdaqhaaWcbaGaemOqaieabaGaeGOmaidaaaqabaGccqGGSaalaaa@5D10@

and its distance to point A is given similarly by

r″=r2−2rcos⁡θxA+xA2=r2+2rcos⁡θxB+xB2.
 MathType@MTEF@5@5@+=feaafiart1ev1aaatCvAUfKttLearuWrP9MDH5MBPbIqV92AaeXatLxBI9gBaebbnrfifHhDYfgasaacH8akY=wiFfYdH8Gipec8Eeeu0xXdbba9frFj0=OqFfea0dXdd9vqai=hGuQ8kuc9pgc9s8qqaq=dirpe0xb9q8qiLsFr0=vr0=vr0dc8meaabaqaciaacaGaaeqabaqabeGadaaakeaacuWGYbGCgaGbaiabg2da9maakaaabaGaemOCai3aaWbaaSqabeaacqaIYaGmaaGccqGHsislcqaIYaGmcqWGYbGCcyGGJbWycqGGVbWBcqGGZbWCiiGacqWF4oqCcqWG4baEdaWgaaWcbaGaemyqaeeabeaakiabgUcaRiabdIha4naaDaaaleaacqWGbbqqaeaacqaIYaGmaaaabeaakiabg2da9maakaaabaGaemOCai3aaWbaaSqabeaacqaIYaGmaaGccqGHRaWkcqaIYaGmcqWGYbGCcyGGJbWycqGGVbWBcqGGZbWCcqWF4oqCcqWG4baEdaWgaaWcbaGaemOqaieabeaakiabgUcaRiabdIha4naaDaaaleaacqWGcbGqaeaacqaIYaGmaaaabeaakiabc6caUaaa@573B@

We are interested in letting *r *go to infinity while keeping *θ *constant. This means that we are considering a point moving away from the two sources in a given direction. The Taylor expansions of expressions *r' *and *r" *can then be obtained as:

r′=r(1−cos⁡θxBr+o(r−1)) and r″=r(1+cos⁡θxBr+o(r−1)),
 MathType@MTEF@5@5@+=feaafiart1ev1aaatCvAUfKttLearuWrP9MDH5MBPbIqV92AaeXatLxBI9gBaebbnrfifHhDYfgasaacH8akY=wiFfYdH8Gipec8Eeeu0xXdbba9frFj0=OqFfea0dXdd9vqai=hGuQ8kuc9pgc9s8qqaq=dirpe0xb9q8qiLsFr0=vr0=vr0dc8meaabaqaciaacaGaaeqabaqabeGadaaakeaacuWGYbGCgaqbaiabg2da9iabdkhaYnaabmaabaGaeGymaeJaeyOeI0YaaSaaaeaacyGGJbWycqGGVbWBcqGGZbWCiiGacqWF4oqCcqWG4baEdaWgaaWcbaGaemOqaieabeaaaOqaaiabdkhaYbaacqGHRaWkcqqGVbWBdaqadaqaaiabdkhaYnaaCaaaleqabaGaeyOeI0IaeGymaedaaaGccaGLOaGaayzkaaaacaGLOaGaayzkaaGaeeiiaaIaeeyyaeMaeeOBa4MaeeizaqMaeeiiaaIafmOCaiNbayaacqGH9aqpcqWGYbGCdaqadaqaaiabigdaXiabgUcaRmaalaaabaGagi4yamMaei4Ba8Maei4CamNae8hUdeNaemiEaG3aaSbaaSqaaiabdkeacbqabaaakeaacqWGYbGCaaGaey4kaSIaee4Ba82aaeWaaeaacqWGYbGCdaahaaWcbeqaaiabgkHiTiabigdaXaaaaOGaayjkaiaawMcaaaGaayjkaiaawMcaaiabcYcaSaaa@6479@

where *o*(*r*^-1^) stands for any function negligible compared to *r*^-1 ^when *r *tends to +∞.

The ratio of propagules from B over propagules from A at point (*x, y*) = (*r*, *θ*) can then be written as

ρB(r,θ)=γr(r′)γr(r″)=γr(r−cos⁡θxB+o(1))γr(r+cos⁡θxB+o(1)),
 MathType@MTEF@5@5@+=feaafiart1ev1aaatCvAUfKttLearuWrP9MDH5MBPbIqV92AaeXatLxBI9gBaebbnrfifHhDYfgasaacH8akY=wiFfYdH8Gipec8Eeeu0xXdbba9frFj0=OqFfea0dXdd9vqai=hGuQ8kuc9pgc9s8qqaq=dirpe0xb9q8qiLsFr0=vr0=vr0dc8meaabaqaciaacaGaaeqabaqabeGadaaakeaaiiGacqWFbpGCdaWgaaWcbaGaemOqaieabeaakiabcIcaOiabdkhaYjabcYcaSiab=H7aXjabcMcaPiabg2da9maalaaabaGae83SdC2aaSbaaSqaaiabdkhaYbqabaGccqGGOaakcuWGYbGCgaqbaiabcMcaPaqaaiab=n7aNnaaBaaaleaacqWGYbGCaeqaaOGaeiikaGIafmOCaiNbayaacqGGPaqkaaGaeyypa0ZaaSaaaeaacqWFZoWzdaWgaaWcbaGaemOCaihabeaakmaabmaabaGaemOCaiNaeyOeI0Iagi4yamMaei4Ba8Maei4CamNae8hUdeNaemiEaG3aaSbaaSqaaiabdkeacbqabaGccqGHRaWkcqqGVbWBcqGGOaakcqaIXaqmcqGGPaqkaiaawIcacaGLPaaaaeaacqWFZoWzdaWgaaWcbaGaemOCaihabeaakmaabmaabaGaemOCaiNaey4kaSIagi4yamMaei4Ba8Maei4CamNae8hUdeNaemiEaG3aaSbaaSqaaiabdkeacbqabaGccqGHRaWkcqqGVbWBcqGGOaakcqaIXaqmcqGGPaqkaiaawIcacaGLPaaaaaGaeiilaWcaaa@6E8C@

where *o*(*1*) stands for any function tending to 0 when *r *tends to +∞.

By analogy with the 1-dimensional equation, in the direction *θ *when *r *tends to +∞, the ratio *ρ*_*B*_(*r*, *θ*) behaves just as the ratio for a 1-dimensional kernel *γ*_*r*_, and a distance between the two sources of 2cos*θx*_*B*_. The following results are thus direct consequences of the 1-dimensional results.

#### Exponential kernels

For an exponential dispersal kernel (see Table 2) the ratio *ρ*_*B*_(*r*, *θ*) tends to exp⁡(2xCαcos⁡θ)
 MathType@MTEF@5@5@+=feaafiart1ev1aaatCvAUfKttLearuWrP9MDH5MBPbIqV92AaeXatLxBI9gBaebbnrfifHhDYfgasaacH8akY=wiFfYdH8Gipec8Eeeu0xXdbba9frFj0=OqFfea0dXdd9vqai=hGuQ8kuc9pgc9s8qqaq=dirpe0xb9q8qiLsFr0=vr0=vr0dc8meaabaqaciaacaGaaeqabaqabeGadaaakeaacyGGLbqzcqGG4baEcqGGWbaCdaqadaqaaiabikdaYmaalaaabaGaemiEaG3aaSbaaSqaaiabdoeadbqabaaakeaaiiGacqWFXoqyaaGagi4yamMaei4Ba8Maei4CamNae8hUdehacaGLOaGaayzkaaaaaa@3DA4@ which is more than 1 for -*π*/2<*θ*<*π*/2 (that is in the direction of B) and less than 1 for *π*/2<*θ*<3*π*/2 (that is in the direction of A). As a result, the proportion of propagules from A, *π*_*A*_(*r*, *θ*) tends to *π*_*lim*_(*θ*), which is between 0 and 1/2 for -*π*/2<*θ*<*π*/2 and between 1/2 and 1 for *π*/2<*θ*<3*π*/2.

#### Exponential power kernels

For an exponential power dispersal kernel (Table 2), if *c*>1,*ρ*_*B*_(*r*, *θ*) tends to +∞ when *π*/2<*θ*<3*π*/2 and tends to -∞ when *π*/2<*θ*<3*π*/2. If *c*<1, *ρ*_*B*_(*r*, *θ*) tends to 1 whatever the value of *θ*. Finally, if *c *= 1, corresponding to the exponential kernel, we find again the previous result: *ρ*_*B*_(*r*, *θ*) tends to exp⁡(2xBαcos⁡θ)
 MathType@MTEF@5@5@+=feaafiart1ev1aaatCvAUfKttLearuWrP9MDH5MBPbIqV92AaeXatLxBI9gBaebbnrfifHhDYfgasaacH8akY=wiFfYdH8Gipec8Eeeu0xXdbba9frFj0=OqFfea0dXdd9vqai=hGuQ8kuc9pgc9s8qqaq=dirpe0xb9q8qiLsFr0=vr0=vr0dc8meaabaqaciaacaGaaeqabaqabeGadaaakeaacyGGLbqzcqGG4baEcqGGWbaCdaqadaqaaiabikdaYmaalaaabaGaemiEaG3aaSbaaSqaaiabdoeadbqabaaakeaaiiGacqWFXoqyaaGagi4yamMaei4Ba8Maei4CamNae8hUdehacaGLOaGaayzkaaaaaa@3DA4@. Consequently, the proportion of propagules from A, *π*_*A*_(*r*, *θ*) tends to 0 for *π*/2<*θ*<3*π*/2 (direction of B) and to 1 for *π*/2<*θ*<3*π*/2 (direction of B) when c>1; it tends to 1/2 whatever the direction *θ *when c<1. For the exponential kernel (c= 1), *π*_*A*_(*r*, *θ*) tends to values strictly between 0 and 1, such as already mentioned above.

#### Power-law kernels

For the power-law kernels, *ρ*_*B*_(*r*, *θ*) tends to 1 for all *a*>2, and all *θ*. This means that the proportion *π*_*A*_(*r*, *θ*) tends to 1/2 in all directions for any power-law kernel.

### Validity of the asymptotic results for natural scales

We computed distances actually travelled by some propagules to evaluate whether the asymptotic results analytically derived are effective at finite distances of biological interest. The range of distances of interest depends on the dispersal kernel, by virtue of the mean distance travelled and the dispersal tail; in addition the range of distances is also very sensitive to the number of propagules *R *emitted by each source [5].

We derived numerically for different kernels *γ *and number of propagules *R*, the distribution of the position of the furthest forward propagule (FFP) coming from B [5]. The probability density function (PDF) of this random variable is given by

*FFP*_*B*_(*x*) = *Rγ*(*x *- *x*_*B*_)*F*(*x *- *x*_*B*_)^*R*-1^

where *F *is the cumulative distribution function (CDF) associated with *γ*. This expression means (i) that the further forward propagule from B is one of the *R *propagules emitted by B, (ii) that it falls at a distance *x-x*_*B *_from its source (B) and (iii) that the *R-1 *other propagules from B fall at distances smaller than (*x-x*_*B*_).

As a simplification, we have chosen to focus on the position of the FFP from B (and not on the position of the FFP from both sources A and B), because its distribution does not depend on the position of source A. This is a conservative choice because the extreme dispersal event of interest for biological questions (such as speed of colonization or contamination between fields...) is the FFP, which is either the FFP from B, or the FFP from A if it travelled further than the FFP from B [5].

We considered three wide-ranged values for R equal to 10; 1000 and 1000000. These orders of magnitude correspond for instance to the numbers of dispersed rodents offspring, tree seeds and crop pollen grains.

The computation of the proportions of propagules from the source A at distances close to that of the FFP from B allowed asserting if the asymptotic properties obtained when the distance is tending toward infinity are good approximations for what happens to the further forward individuals.

## Authors' contributions

EKK, CL and PHG together developed the idea at the origin of the manuscript and discussed its consequences. EKK formalized it mathematically. EKK, CL and PHG cooperated in writing the manuscript and read and approved the final manuscript.

## Supplementary Material

Additional File 1 In the additional file, we provide a general classification of dispersal kernels regarding the amount of long-distance dispersal and a formal proof of the 'mixing' property of long-tailed kernels (a large subset of heavy-tailed kernels) general to all families of kernels. Click here for file

## Figures and Tables

**Table 1:** Six families of 1-dimensional dispersal kernels used in this study, together with their characteristics. The mean distance travelled is obtained from δ=∫−∞+∞|x|γ(x)dx
MathType@MTEF@5@5@+=feaafiart1ev1aaatCvAUfKttLearuWrP9MDH5MBPbIqV92AaeXatLxBI9gBaebbnrfifHhDYfgasaacH8akY=wiFfYdH8Gipec8Eeeu0xXdbba9frFj0=OqFfea0dXdd9vqai=hGuQ8kuc9pgc9s8qqaq=dirpe0xb9q8qiLsFr0=vr0=vr0dc8meaabaqaciaacaGaaeqabaqabeGadaaakeaaiiGacqWF0oazcqGH9aqpdaWdXbqaamaaemaabaGaemiEaGhacaGLhWUaayjcSdGae83SdCMaeiikaGIaemiEaGNaeiykaKIaemizaqMaemiEaGhaleaacqGHsislcqGHEisPaeaacqGHRaWkcqGHEisPa0Gaey4kIipaaaa@42AA@. Expression Γ() stands for the Gamma function.

Kernel families	Expression	Parameters values	Weight of the tail	Mean distance travelled, *δ*
Exponential	γ1(x)=12αexp⁡(−|xα|) MathType@MTEF@5@5@+=feaafiart1ev1aaatCvAUfKttLearuWrP9MDH5MBPbIqV92AaeXatLxBI9gBaebbnrfifHhDYfgasaacH8akY=wiFfYdH8Gipec8Eeeu0xXdbba9frFj0=OqFfea0dXdd9vqai=hGuQ8kuc9pgc9s8qqaq=dirpe0xb9q8qiLsFr0=vr0=vr0dc8meaabaqaciaacaGaaeqabaqabeGadaaakeaaiiGacqWFZoWzdaWgaaWcbaGaeGymaedabeaakiabcIcaOiabdIha4jabcMcaPiabg2da9maalaaabaGaeGymaedabaGaeGOmaiJae8xSdegaaiGbcwgaLjabcIha4jabcchaWnaabmaabaGaeyOeI0YaaqWaaeaadaWcaaqaaiabdIha4bqaaiab=f7aHbaaaiaawEa7caGLiWoaaiaawIcacaGLPaaaaaa@442C@	*α*>0	Rapidly varying Exponential	*α*
Gaussian	γ2(x)=1παexp⁡(−x2α2) MathType@MTEF@5@5@+=feaafiart1ev1aaatCvAUfKttLearuWrP9MDH5MBPbIqV92AaeXatLxBI9gBaebbnrfifHhDYfgasaacH8akY=wiFfYdH8Gipec8Eeeu0xXdbba9frFj0=OqFfea0dXdd9vqai=hGuQ8kuc9pgc9s8qqaq=dirpe0xb9q8qiLsFr0=vr0=vr0dc8meaabaqaciaacaGaaeqabaqabeGadaaakeaaiiGacqWFZoWzdaWgaaWcbaGaeGOmaidabeaakiabcIcaOiabdIha4jabcMcaPiabg2da9maalaaabaGaeGymaedabaWaaOaaaeaacqWFapaCaSqabaGccqWFXoqyaaGagiyzauMaeiiEaGNaeiiCaa3aaeWaaeaacqGHsisldaWcaaqaaiabdIha4naaCaaaleqabaGaeGOmaidaaaGcbaGae8xSde2aaWbaaSqabeaacqaIYaGmaaaaaaGccaGLOaGaayzkaaaaaa@4449@	*α*>0	Rapidly varying Thin-tailed	απ MathType@MTEF@5@5@+=feaafiart1ev1aaatCvAUfKttLearuWrP9MDH5MBPbIqV92AaeXatLxBI9gBaebbnrfifHhDYfgasaacH8akY=wiFfYdH8Gipec8Eeeu0xXdbba9frFj0=OqFfea0dXdd9vqai=hGuQ8kuc9pgc9s8qqaq=dirpe0xb9q8qiLsFr0=vr0=vr0dc8meaabaqaciaacaGaaeqabaqabeGadaaakeaadaWcaaqaaGGaciab=f7aHbqaamaakaaabaGae8hWdahaleqaaaaaaaa@3035@
Power-law	γ3(x)=a−12α(1+|xα|)−a MathType@MTEF@5@5@+=feaafiart1ev1aaatCvAUfKttLearuWrP9MDH5MBPbIqV92AaeXatLxBI9gBaebbnrfifHhDYfgasaacH8akY=wiFfYdH8Gipec8Eeeu0xXdbba9frFj0=OqFfea0dXdd9vqai=hGuQ8kuc9pgc9s8qqaq=dirpe0xb9q8qiLsFr0=vr0=vr0dc8meaabaqaciaacaGaaeqabaqabeGadaaakeaaiiGacqWFZoWzdaWgaaWcbaGaeG4mamdabeaakiabcIcaOiabdIha4jabcMcaPiabg2da9maalaaabaGaemyyaeMaeyOeI0IaeGymaedabaGaeGOmaiJae8xSdegaamaabmaabaGaeGymaeJaey4kaSYaaqWaaeaadaWcaaqaaiabdIha4bqaaiab=f7aHbaaaiaawEa7caGLiWoaaiaawIcacaGLPaaadaahaaWcbeqaaiabgkHiTiabdggaHbaaaaa@457E@	*α*>0 *a*>1	Regularly varying Fat-tailed	αa−2 MathType@MTEF@5@5@+=feaafiart1ev1aaatCvAUfKttLearuWrP9MDH5MBPbIqV92AaeXatLxBI9gBaebbnrfifHhDYfgasaacH8akY=wiFfYdH8Gipec8Eeeu0xXdbba9frFj0=OqFfea0dXdd9vqai=hGuQ8kuc9pgc9s8qqaq=dirpe0xb9q8qiLsFr0=vr0=vr0dc8meaabaqaciaacaGaaeqabaqabeGadaaakeaadaWcaaqaaGGaciab=f7aHbqaaiabdggaHjabgkHiTiabikdaYaaaaaa@318C@
Exponential power	γ4(x)=c2αΓ(1/c)exp⁡(−|xα|c) MathType@MTEF@5@5@+=feaafiart1ev1aaatCvAUfKttLearuWrP9MDH5MBPbIqV92AaeXatLxBI9gBaebbnrfifHhDYfgasaacH8akY=wiFfYdH8Gipec8Eeeu0xXdbba9frFj0=OqFfea0dXdd9vqai=hGuQ8kuc9pgc9s8qqaq=dirpe0xb9q8qiLsFr0=vr0=vr0dc8meaabaqaciaacaGaaeqabaqabeGadaaakeaaiiGacqWFZoWzdaWgaaWcbaGaeGinaqdabeaakiabcIcaOiabdIha4jabcMcaPiabg2da9maalaaabaGaem4yamgabaGaeGOmaiJae8xSdeMaeu4KdCKaeiikaGIaeGymaeJaei4la8Iaem4yamMaeiykaKcaaiGbcwgaLjabcIha4jabcchaWnaabmaabaGaeyOeI0YaaqWaaeaadaWcaaqaaiabdIha4bqaaiab=f7aHbaaaiaawEa7caGLiWoadaahaaWcbeqaaiabdogaJbaaaOGaayjkaiaawMcaaaaa@4C56@	*α*,*c*>0	Rapidly varying Thin-tailed for c>1 Fat-tailed for c<1 Exponential for c = 1	αΓ(2/c)Γ(1/c) MathType@MTEF@5@5@+=feaafiart1ev1aaatCvAUfKttLearuWrP9MDH5MBPbIqV92AaeXatLxBI9gBaebbnrfifHhDYfgasaacH8akY=wiFfYdH8Gipec8Eeeu0xXdbba9frFj0=OqFfea0dXdd9vqai=hGuQ8kuc9pgc9s8qqaq=dirpe0xb9q8qiLsFr0=vr0=vr0dc8meaabaqaciaacaGaaeqabaqabeGadaaakeaaiiGacqWFXoqydaWcaaqaaiabfo5ahjabcIcaOiabikdaYiabc+caViabdogaJjabcMcaPaqaaiabfo5ahjabcIcaOiabigdaXiabc+caViabdogaJjabcMcaPaaaaaa@3AE2@
2Dt	γ5(x)=Γ(a)απΓ(a−12)(1+x2α2)−a MathType@MTEF@5@5@+=feaafiart1ev1aaatCvAUfKttLearuWrP9MDH5MBPbIqV92AaeXatLxBI9gBaebbnrfifHhDYfgasaacH8akY=wiFfYdH8Gipec8Eeeu0xXdbba9frFj0=OqFfea0dXdd9vqai=hGuQ8kuc9pgc9s8qqaq=dirpe0xb9q8qiLsFr0=vr0=vr0dc8meaabaqaciaacaGaaeqabaqabeGadaaakeaaiiGacqWFZoWzdaWgaaWcbaGaeGynaudabeaakiabcIcaOiabdIha4jabcMcaPiabg2da9maalaaabaGaeu4KdCKaeiikaGIaemyyaeMaeiykaKcabaGae8xSde2aaOaaaeaacqWFapaCaSqabaGccqqHtoWrdaqadaqaaiabdggaHjabgkHiTmaalaaabaGaeGymaedabaGaeGOmaidaaaGaayjkaiaawMcaaaaadaqadaqaaiabigdaXiabgUcaRmaalaaabaGaemiEaG3aaWbaaSqabeaacqaIYaGmaaaakeaacqWFXoqydaahaaWcbeqaaiabikdaYaaaaaaakiaawIcacaGLPaaadaahaaWcbeqaaiabgkHiTiabdggaHbaaaaa@4DF5@	*α*>0 *a*>1/2	Regularly varying Fat-tailed	απ(a−1)Γ(a)Γ(a−12) MathType@MTEF@5@5@+=feaafiart1ev1aaatCvAUfKttLearuWrP9MDH5MBPbIqV92AaeXatLxBI9gBaebbnrfifHhDYfgasaacH8akY=wiFfYdH8Gipec8Eeeu0xXdbba9frFj0=OqFfea0dXdd9vqai=hGuQ8kuc9pgc9s8qqaq=dirpe0xb9q8qiLsFr0=vr0=vr0dc8meaabaqaciaacaGaaeqabaqabeGadaaakeaadaWcaaqaaGGaciab=f7aHbqaamaakaaabaGae8hWdahaleqaaOGaeiikaGIaemyyaeMaeyOeI0IaeGymaeJaeiykaKcaamaalaaabaGaeu4KdCKaeiikaGIaemyyaeMaeiykaKcabaGaeu4KdC0aaeWaaeaacqWGHbqycqGHsisldaWcaaqaaiabigdaXaqaaiabikdaYaaaaiaawIcacaGLPaaaaaaaaa@40A9@
Mixture of two Gaussians	γ6(x)=p(1πα1e−x2/α12)+(1−p)(1πα2e−x2/α22) MathType@MTEF@5@5@+=feaafiart1ev1aaatCvAUfKttLearuWrP9MDH5MBPbIqV92AaeXatLxBI9gBaebbnrfifHhDYfgasaacH8akY=wiFfYdH8Gipec8Eeeu0xXdbba9frFj0=OqFfea0dXdd9vqai=hGuQ8kuc9pgc9s8qqaq=dirpe0xb9q8qiLsFr0=vr0=vr0dc8meaabaqaciaacaGaaeqabaqabeGadaaakeaaiiGacqWFZoWzdaWgaaWcbaGaeGOnaydabeaakiabcIcaOiabdIha4jabcMcaPiabg2da9iabdchaWnaabmaabaWaaSaaaeaacqaIXaqmaeaadaGcaaqaaiab=b8aWbWcbeaakiab=f7aHnaaBaaaleaacqaIXaqmaeqaaaaakiabdwgaLnaaCaaaleqabaWaaSGbaeaacqGHsislcqWG4baEdaahaaadbeqaaiabikdaYaaaaSqaaiab=f7aHnaaDaaameaacqaIXaqmaeaacqaIYaGmaaaaaaaaaOGaayjkaiaawMcaaiabgUcaRmaabmaabaGaeGymaeJaeyOeI0IaemiCaahacaGLOaGaayzkaaWaaeWaaeaadaWcaaqaaiabigdaXaqaamaakaaabaGae8hWdahaleqaaOGae8xSde2aaSbaaSqaaiabikdaYaqabaaaaOGaemyzau2aaWbaaSqabeaadaWcgaqaaiabgkHiTiabdIha4naaCaaameqabaGaeGOmaidaaaWcbaGae8xSde2aa0baaWqaaiabikdaYaqaaiabikdaYaaaaaaaaaGccaGLOaGaayzkaaaaaa@5ADB@	*α*_1_≠*α*_2_ *p*>0	Rapidly varying Thin-tailed (but leptokurtic)	pα1π+(1−p)α2π MathType@MTEF@5@5@+=feaafiart1ev1aaatCvAUfKttLearuWrP9MDH5MBPbIqV92AaeXatLxBI9gBaebbnrfifHhDYfgasaacH8akY=wiFfYdH8Gipec8Eeeu0xXdbba9frFj0=OqFfea0dXdd9vqai=hGuQ8kuc9pgc9s8qqaq=dirpe0xb9q8qiLsFr0=vr0=vr0dc8meaabaqaciaacaGaaeqabaqabeGadaaakeaacqWGWbaCdaWcaaqaaGGaciab=f7aHnaaBaaaleaacqaIXaqmaeqaaaGcbaWaaOaaaeaacqWFapaCaSqabaaaaOGaey4kaSYaaeWaaeaacqaIXaqmcqGHsislcqWGWbaCaiaawIcacaGLPaaadaWcaaqaaiab=f7aHnaaBaaaleaacqaIYaGmaeqaaaGcbaWaaOaaaeaacqWFapaCaSqabaaaaaaa@3D24@

**Table 2:** Four families of 2-dimensional dispersal kernels used in this study, together with their characteristics. The mean distance travelled is obtained from δ=∫0+∞γ(r)2πrdr
MathType@MTEF@5@5@+=feaafiart1ev1aaatCvAUfKttLearuWrP9MDH5MBPbIqV92AaeXatLxBI9gBaebbnrfifHhDYfgasaacH8akY=wiFfYdH8Gipec8Eeeu0xXdbba9frFj0=OqFfea0dXdd9vqai=hGuQ8kuc9pgc9s8qqaq=dirpe0xb9q8qiLsFr0=vr0=vr0dc8meaabaqaciaacaGaaeqabaqabeGadaaakeaaiiGacqWF0oazcqGH9aqpdaWdXbqaaiab=n7aNjabcIcaOiabdkhaYjabcMcaPiabikdaYiab=b8aWjabdkhaYjabdsgaKjabdkhaYbWcbaGaeGimaadabaGaey4kaSIaeyOhIukaniabgUIiYdaaaa@409E@. Expression Γ() stands for the Gamma function.

Kernel families	Expression	Parameters values	Weight of the tail	Mean distance travelled, *δ*
Exponential	γ1(x,y)=12πα2exp⁡(−rα) MathType@MTEF@5@5@+=feaafiart1ev1aaatCvAUfKttLearuWrP9MDH5MBPbIqV92AaeXatLxBI9gBaebbnrfifHhDYfgasaacH8akY=wiFfYdH8Gipec8Eeeu0xXdbba9frFj0=OqFfea0dXdd9vqai=hGuQ8kuc9pgc9s8qqaq=dirpe0xb9q8qiLsFr0=vr0=vr0dc8meaabaqaciaacaGaaeqabaqabeGadaaakeaaiiGacqWFZoWzdaWgaaWcbaGaeGymaedabeaakiabcIcaOiabdIha4jabcYcaSiabdMha5jabcMcaPiabg2da9maalaaabaGaeGymaedabaGaeGOmaiJae8hWdaNae8xSde2aaWbaaSqabeaacqaIYaGmaaaaaOGagiyzauMaeiiEaGNaeiiCaa3aaeWaaeaacqGHsisldaWcaaqaaiabdkhaYbqaaiab=f7aHbaaaiaawIcacaGLPaaaaaa@463A@	*α *>0	Rapidly varying Exponential	2*α*
Gaussian	γ2(x,y)=1πα2exp⁡(−x2+y2α2) MathType@MTEF@5@5@+=feaafiart1ev1aaatCvAUfKttLearuWrP9MDH5MBPbIqV92AaeXatLxBI9gBaebbnrfifHhDYfgasaacH8akY=wiFfYdH8Gipec8Eeeu0xXdbba9frFj0=OqFfea0dXdd9vqai=hGuQ8kuc9pgc9s8qqaq=dirpe0xb9q8qiLsFr0=vr0=vr0dc8meaabaqaciaacaGaaeqabaqabeGadaaakeaaiiGacqWFZoWzdaWgaaWcbaGaeGOmaidabeaakiabcIcaOiabdIha4jabcYcaSiabdMha5jabcMcaPiabg2da9maalaaabaGaeGymaedabaGae8hWdaNae8xSde2aaWbaaSqabeaacqaIYaGmaaaaaOGagiyzauMaeiiEaGNaeiiCaa3aaeWaaeaacqGHsisldaWcaaqaaiabdIha4naaCaaaleqabaGaeGOmaidaaOGaey4kaSIaemyEaK3aaWbaaSqabeaacqaIYaGmaaaakeaacqWFXoqydaahaaWcbeqaaGqaaiab+jdaYaaaaaaakiaawIcacaGLPaaaaaa@4B2D@	*α *>0	Rapidly varying Thin-tailed	απ2 MathType@MTEF@5@5@+=feaafiart1ev1aaatCvAUfKttLearuWrP9MDH5MBPbIqV92AaeXatLxBI9gBaebbnrfifHhDYfgasaacH8akY=wiFfYdH8Gipec8Eeeu0xXdbba9frFj0=OqFfea0dXdd9vqai=hGuQ8kuc9pgc9s8qqaq=dirpe0xb9q8qiLsFr0=vr0=vr0dc8meaabaqaciaacaGaaeqabaqabeGadaaakeaadaWcaaqaaGGaciab=f7aHnaakaaabaGae8hWdahaleqaaaGcbaGaeGOmaidaaaaa@3131@
Power-law	γ3(x,y)=(a−2)(a−1)2πα2(1+rα)−a MathType@MTEF@5@5@+=feaafiart1ev1aaatCvAUfKttLearuWrP9MDH5MBPbIqV92AaeXatLxBI9gBaebbnrfifHhDYfgasaacH8akY=wiFfYdH8Gipec8Eeeu0xXdbba9frFj0=OqFfea0dXdd9vqai=hGuQ8kuc9pgc9s8qqaq=dirpe0xb9q8qiLsFr0=vr0=vr0dc8meaabaqaciaacaGaaeqabaqabeGadaaakeaaiiGacqWFZoWzdaWgaaWcbaGaeG4mamdabeaakiabcIcaOiabdIha4jabcYcaSiabdMha5jabcMcaPiabg2da9maalaaabaGaeiikaGIaemyyaeMaeyOeI0IaeGOmaiJaeiykaKIaeiikaGIaemyyaeMaeyOeI0IaeGymaeJaeiykaKcabaGaeGOmaiJae8hWdaNae8xSde2aaWbaaSqabeaacqaIYaGmaaaaaOWaaeWaaeaacqaIXaqmcqGHRaWkdaWcaaqaaiabdkhaYbqaaiab=f7aHbaaaiaawIcacaGLPaaadaahaaWcbeqaaiabgkHiTiabdggaHbaaaaa@4E1A@	*α*>0 *a*>2	Regularly varying Fat-tailed	2αa−3 MathType@MTEF@5@5@+=feaafiart1ev1aaatCvAUfKttLearuWrP9MDH5MBPbIqV92AaeXatLxBI9gBaebbnrfifHhDYfgasaacH8akY=wiFfYdH8Gipec8Eeeu0xXdbba9frFj0=OqFfea0dXdd9vqai=hGuQ8kuc9pgc9s8qqaq=dirpe0xb9q8qiLsFr0=vr0=vr0dc8meaabaqaciaacaGaaeqabaqabeGadaaakeaadaWcaaqaaiabikdaYGGaciab=f7aHbqaaiabdggaHjabgkHiTiabiodaZaaaaaa@3280@
Exponential power	γ4(x,y)=c2πα2Γ(2/c)exp⁡(−(rα)c) MathType@MTEF@5@5@+=feaafiart1ev1aaatCvAUfKttLearuWrP9MDH5MBPbIqV92AaeXatLxBI9gBaebbnrfifHhDYfgasaacH8akY=wiFfYdH8Gipec8Eeeu0xXdbba9frFj0=OqFfea0dXdd9vqai=hGuQ8kuc9pgc9s8qqaq=dirpe0xb9q8qiLsFr0=vr0=vr0dc8meaabaqaciaacaGaaeqabaqabeGadaaakeaaiiGacqWFZoWzdaWgaaWcbaGaeGinaqdabeaakiabcIcaOiabdIha4jabcYcaSiabdMha5jabcMcaPiabg2da9maalaaabaGaem4yamgabaGaeGOmaiJae8hWdaNae8xSde2aaWbaaSqabeaacqaIYaGmaaGccqqHtoWrcqGGOaakcqaIYaGmcqGGVaWlcqWGJbWycqGGPaqkaaGagiyzauMaeiiEaGNaeiiCaa3aaeWaaeaacqGHsisldaqadaqaamaalaaabaGaemOCaihabaGae8xSdegaaaGaayjkaiaawMcaamaaCaaaleqabaGaem4yamgaaaGccaGLOaGaayzkaaaaaa@4FEF@	*α*,*c*>0	Rapidly varying Thin-tailed for c>1 Fat-tailed for c<1 Exponential for c = 1	αΓ(3/c)Γ(2/c) MathType@MTEF@5@5@+=feaafiart1ev1aaatCvAUfKttLearuWrP9MDH5MBPbIqV92AaeXatLxBI9gBaebbnrfifHhDYfgasaacH8akY=wiFfYdH8Gipec8Eeeu0xXdbba9frFj0=OqFfea0dXdd9vqai=hGuQ8kuc9pgc9s8qqaq=dirpe0xb9q8qiLsFr0=vr0=vr0dc8meaabaqaciaacaGaaeqabaqabeGadaaakeaaiiGacqWFXoqydaWcaaqaaiabfo5ahjabcIcaOiabiodaZiabc+caViabdogaJjabcMcaPaqaaiabfo5ahjabcIcaOiabikdaYiabc+caViabdogaJjabcMcaPaaaaaa@3AE6@
