# Supplementary material for: Mixing of propagules from discrete sources at long distance: comparing a dispersal tail to an exponential
Source: BMC Ecol. 2006 Feb 20;6:3. doi: 10.1186/1472-6785-6-3 (PMC1450262; doi:10.1186/1472-6785-6-3)
Supplement: Additional File 1 — In the additional file, we provide a general classification of dispersal kernels regarding the amount of long-distance dispersal and a formal proof of the 'mixing' property of long-tailed kernels (a large subset of heavy-tailed kernels) general to all families of kernels. [file 1472-6785-6-3-S1.pdf]

Classification of dispersal tails and general result concerning long-distance mixing of propagules

The vocabulary concerning heavy tails may appear confusing in the literature because (i) no strict definition of "heavy tailed distributions" seems to exist and (ii) the different fields of application (ruin theory, time series…) do not require the same types of heavy-tailed distributions. We present here several classes of heavy tailed distributions. They are classically defined by characterizing the tail of the cumulative distribution function ($\overline{\Gamma}(x) = 1 - \Gamma(x) = 1 - \int_0^x \gamma(y)dy$) and we adapted the definitions to the tail of the density functions (dispersal kernel are supposed to be always decreasing to 0). For more details, see [1].

Definitions of some classes of heavy-tailed kernels.

$\mathcal{K}$ is the set of fat-tailed kernels γ. By definition they are the kernels not exponentially bounded, i.e. such that

$$\lim_{x\to\infty} \exp(\varepsilon x)\gamma(x) = \infty, \text{ for all } \varepsilon>0.$$

$\mathcal{L}$ is the set of long-tailed kernels γ , i.e. such that

$$\lim_{x\to\infty} \frac{\gamma(x-y)}{\gamma(x)} = 1, \text{ for all } y\geq 0.$$

$\mathcal{R}_{-\alpha}$ is the set of kernels γ regularly varying with index of variation –α where α is a positive real number, i.e. such that

$$\lim_{x\to\infty} \frac{\gamma(\lambda x)}{\gamma(x)} = \lambda^{-\alpha}, \text{ for all } \lambda\geq 0.$$

Remarks

(i) $\mathcal{R}_{-\alpha} \subset \mathcal{L} \subset \mathcal{K}$ (e.g. Fig. 1.4.1 in [1]) and these are strict inclusions. Few functions belong to $\mathcal{K}$ but not $\mathcal{L}$ (see Section 1.4 in [1] for an example). However, numerous functions, among them many classically used, belong to $\mathcal{L}$ but not to $\mathcal{R}_{-\alpha}$ (e.g. Table 1).

(ii) $\mathcal{K}$ is the set of fat-tailed kernels [2]. For these kernels the moment generating function is undefined [1], leading to a travelling wave of increasing speed [2, 3].

(iii) We can also define $\mathcal{R}_0$ as the set of functions slowly varying, i.e. such that

$$\lim_{x\to\infty} \frac{\gamma(\lambda x)}{\gamma(x)} = 1, \text{ for all } \lambda \geq 0,$$

and $\mathcal{R}_{-\infty}$ as the set of kernels rapidly varying, i.e. such that

$$\lim_{x\to\infty} \frac{\gamma(\lambda x)}{\gamma(x)} = 0, \text{ for all } \lambda > 1.$$

However no dispersal kernel belongs to $\mathcal{R}_0$ because it contains functions with too heavy tails to define probability densities. $\mathcal{R}_{-\infty}$ contains both heavy-tailed kernels and light-tailed kernels and is thus not included in $\mathcal{L}$ or $\mathcal{K}$.

(iv) Regularly varying functions with $\alpha>0$ also exist but are not dispersal kernels since they do not decrease to 0.

(v) The tail of a kernel obtained as a mixture of two kernels has the same type as the heavier tail of the two kernels. Thus mixtures of two Gaussian, classically used as typical leptokurtic kernels (e.g. [4] are thin-tailed. As an opposite example, the kernel

$$0.75 \tfrac{4}{2*10*\Gamma(1/4)} \exp\left(-\left(\tfrac{x}{10}\right)^4\right) + 0.25 \tfrac{0.8}{2*\Gamma(1/0.8)} \exp\left(-x^{0.8}\right),$$

is fat-tailed and rapidly varying because so is the right hand-term. Note however that it is platykurtic ($\frac{\mu_4}{\mu_2^2} = 2.73 < 3$, where μ_4 and μ_2 are the second and fourth moments).

Examples

Regularly varying kernels have the general form $\gamma(r) = r^a\varphi(r)$, with $\varphi(r)$ a slowly varying function, so they gather the power-law functions or geometric decreases.

Fat-tailed kernels include the regularly varying kernels, but also exponential power function with $b<1$ (Table 1).

Slowly varying functions that decrease to 0 are for instance $\log(r)^{-c}$, with $c>0$. Products and sums of slowly varying functions are also slowly varying.

Definitions of some classes of light-tailed kernels.

Among the light-tailed kernels, we distinguish:

$\mathcal{E}$, the set of kernels that are exponential-like, i.e. such that there exist a value $\varepsilon_0>0$ with

$$\lim_{x\to\infty} \exp(\varepsilon x)\gamma(x) = \infty, \text{ for all } \varepsilon>\varepsilon_0 \text{ and } \lim_{x\to\infty} \exp(\varepsilon x)\gamma(x) = 0, \text{ for all } \varepsilon<\varepsilon_0.$$

$\mathcal{T}$, the set of kernels that are bounded by any exponential (thin-tailed), i.e. such that

$$\lim_{x\to\infty} \exp(\varepsilon x)\gamma(x) = 0, \text{ for all } \varepsilon>0.$$

Remarks

(i) The functions belonging to $\mathcal{E}$ are of the general form $\gamma(r) = \exp(-ar)\varphi(r)$, with $\varphi(r)$ a fat-tailed function. They are neither fat-tailed nor thin-tailed.

General results concerning long-distance mixing of propagules

The ratio $\rho_B(x) = \frac{\gamma(x - x_B)}{\gamma(x - x_A)}$ has the same asymptotic behaviour as $\frac{\gamma(x-\delta)}{\gamma(x)} = \frac{\gamma(\log(\lambda y))}{\gamma(\log(y))}$,

where $\delta = x_B - x_A$, $x = \log(y)$ and $-\delta = \log(\lambda)$.

First, note that the ratio $\rho_B(x)$ is constant if and only if γ is exactly an exponential function. The exponential functions are indeed the only ones that verify $f(x+\delta) = f(x)K(\delta)$ for any x and δ (e.g. [5], K being a function not depending on x (that turns to be equal to f up to a multiplicative constant).

Even mixing of propagules- Following the definitions given above, the ratio $\rho_B(x)$ tends toward 1 if and only if γ is a long-tailed kernel, which is not strictly equivalent to a fat-tailed kernel. However, functions belonging to $\mathcal{K}$ but not $\mathcal{L}$ are rare and we are not aware of examples among the classical functions used as dispersal kernels. Although we did not prove it, we expect that functions in $\mathcal{K}$ but not $\mathcal{L}$ are not smooth enough to be dispersal kernels.

Uneven mixing of propagules - $\frac{\gamma(\log(\lambda y))}{\gamma(\log(y))}$ tends to a function $g(\lambda)$ depending on λ, if and only if $g(\lambda) = \lambda^a$ and $\gamma(\log(y))$ is of the form $y^a \varphi(y)$ with $\varphi(y)$ a slowly varying function (e.g. Appendix A3 in [1]. This means that π_A tends to a value strictly between 0 and ½ only for kernels of the form $\gamma(x) = \exp(ax)\varphi(\exp(x))$. When $\varphi(y)$ is any kind of slowly varying function, $\varphi(\exp(x))$ is within the set of long-tailed functions. So here again, we do not find exactly the set of exponential-like functions, but only those of the form $\exp(ax)\varphi(x)$ with $\varphi(x)$ belonging to $\mathcal{L}$.

Absence of mixing of propagules- As above, the ratio $\rho_B(x)$ does not tend towards $+\infty$ for all thin-tailed kernels but for a subset of them (defined, just symmetrically to the long-tailed functions $\mathcal{L}$, as all functions such that $\lim_{x \to \infty} \frac{\gamma(x-y)}{\gamma(x)} = \infty$, for all $y \geq 0$). Here again, although not

proven, we expect that all thin-tailed functions sufficiently smooth to be a dispersal kernel belong to this subset.

Literature Cited

1. Embrechts P, Klüppelberg C, Mikosch T: **Modelling extremal events (for insurance and finance)**. Berlin: Springer-Verlag; 1997.
2. Kot M, Lewis MA, Van Den Driessche P: **Dispersal data and the spread of invading organisms**. *Ecology* 1996, **77**(7):2027-2042.
3. Mollison D: **Spatial contact models for ecological and epidemic spread**. *Journal of the Royal Statistical Society B* 1977, **39**(3):283-326.
4. Le Corre V, Machon N, Petit RJ, Kremer A: **Colonization with long-distance seed dispersal and genetic structure of maternally inherited genes in forest trees: A simulation study**. *Genet Res* 1997, **69**(2):117-125.
5. Rudin W: **Real and complex analysis**. New-York: Mc Graw-Hill; 1966.
